# Beyond the Heel: Unraveling Sever’s Disease and Achilles Tendinitis Through Ultrasound Diagnosis

**DOI:** 10.7759/cureus.54911

**Published:** 2024-02-26

**Authors:** Yassine Nkhili, Yassine Benghali, Abderrahim Lachhab, Ahmed Amine El Oumri

**Affiliations:** 1 Physical Medicine and Rehabilitation, Mohammed First University of Oujda, Oujda, MAR

**Keywords:** tendon injury, comprehensive approach, sever's disease, diagnostic ultrasonography, heel pain

## Abstract

Sever's disease, or calcaneal apophysitis, is a common cause of heel pain in physically active children. This case report presents the evaluation, diagnosis, and management of a 10-year-old female patient with persistent left heel pain. Clinical examination and diagnostic ultrasound confirmed the diagnosis of Sever's disease. Treatment involved a comprehensive approach, including medication, immobilization, therapy modalities, and exercises. The patient showed improvement after 10 weeks of therapy. This case emphasizes the significance of early recognition, accurate diagnosis, and multimodal management for successful outcomes in Sever's disease.

## Introduction

Sever's disease is a prevalent cause of heel pain in physically active children aged eight to 15 years, similar to Osgood-Schlatter disease (OSD). However, unlike OSD, which commonly affects the distal patellar tendon, the involvement of the Achilles tendon and retrocalcaneal bursa is not a consistent feature of Sever's disease [1]. Moreover, it is often undiagnosed by emergency department providers [2].

Sever's disease involves overuse-induced stress on the calcaneus growth plate due to repetitive impact [3]. Also, elevated levels of physical activity and obesity emerge as the predominant risk factors contributing to the development of Sever's disease in pediatric patients [4-5]. Diagnosis relies on clinical evaluation, notably tenderness during heel palpation and compression [3]. Radiographs may support diagnosis. Ultrasound is pivotal for confirmation and assessing inflammation severity, while MRI serves as a valuable tool in assessing the extent and severity of the inflammatory process, providing crucial information for accurate diagnosis and appropriate management of Sever's disease [6-9].

This case presentation aims to shed light on the clinical features of Sever's disease through the examination of a 10-year-old patient with a 12-month history of heel pain.

## Case presentation

A 10-year-old female patient presented to the Department of Physical Medicine and Rehabilitation with a complaint of persistent left heel pain for 12 months. She reported that the pain worsened during the past two weeks and was aggravated by activity while being relieved by rest. The patient mentioned walking on tiptoes to alleviate the pain. There was no history of weight loss, fever, allergies, chronic diseases, hospitalizations, or surgeries. Additionally, no familial history of the disease was reported.

At that time, the physical examination appeared normal, except for mild swelling and localized tenderness observed over the posterior right heel. The patient exhibited signs of obesity, with a significantly elevated body mass index (BMI) of 35, and had a normal and non-pale skin tone. Vital signs were within the normal range. The patient exhibited a limp, indicating sensitivity in the posterior calcanei, particularly on the left side. Furthermore, there was a decreased range of motion for dorsiflexion in the left subtalar joint. Thompson's test yielded normal results.

**Figure 1:**
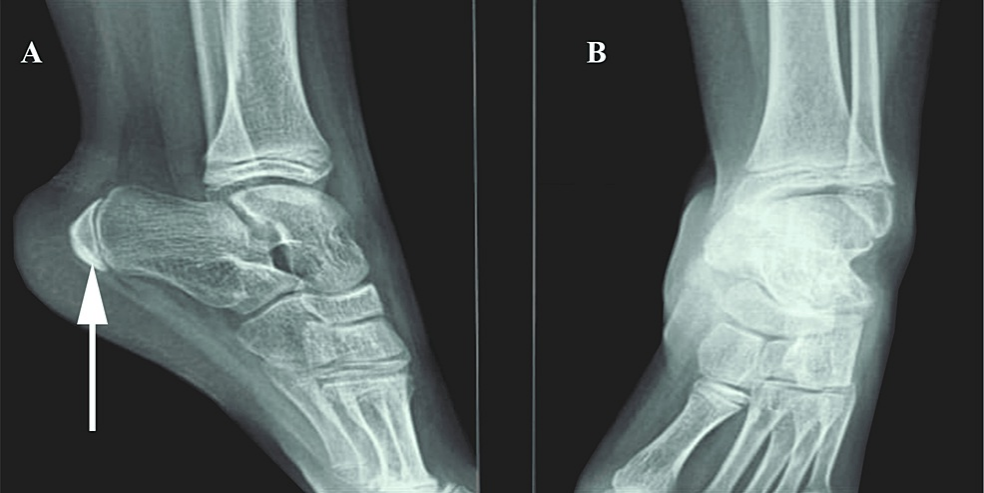
Anteroposterior and lateral radiographs of the left foot demonstrating the presence of calcaneal apophyseal fragmentation (white arrow) associated with increased bone density (A) Lateral X-ray; (B) Posteroanterior X-ray

The patient was advised to undergo regular monitoring of complete blood count, erythrocyte sedimentation rate, alkaline phosphatase, and serum calcium levels. The rheumatoid factor test yielded negative results. X-ray examination of the heel (Figure 1) revealed fragmentation and increased density of the calcaneal apophysis.

**Figure 2:**
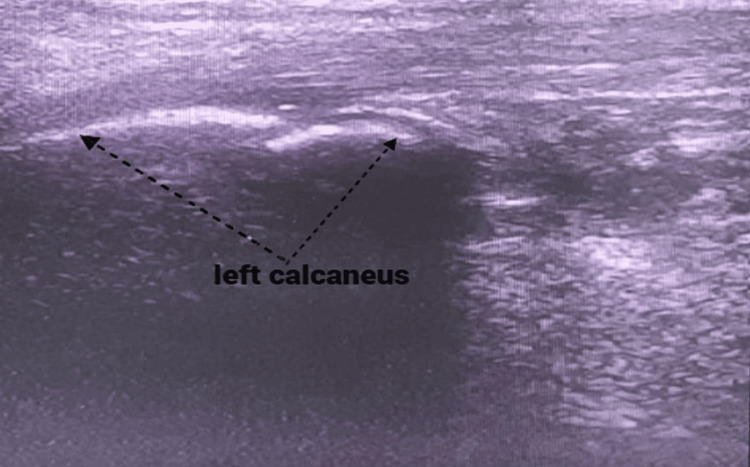
Ultrasound of the left heel longitudinal section showing the presence of fragmentation of the calcaneal apophysis consistent with Sever's disease

**Figure 3:**
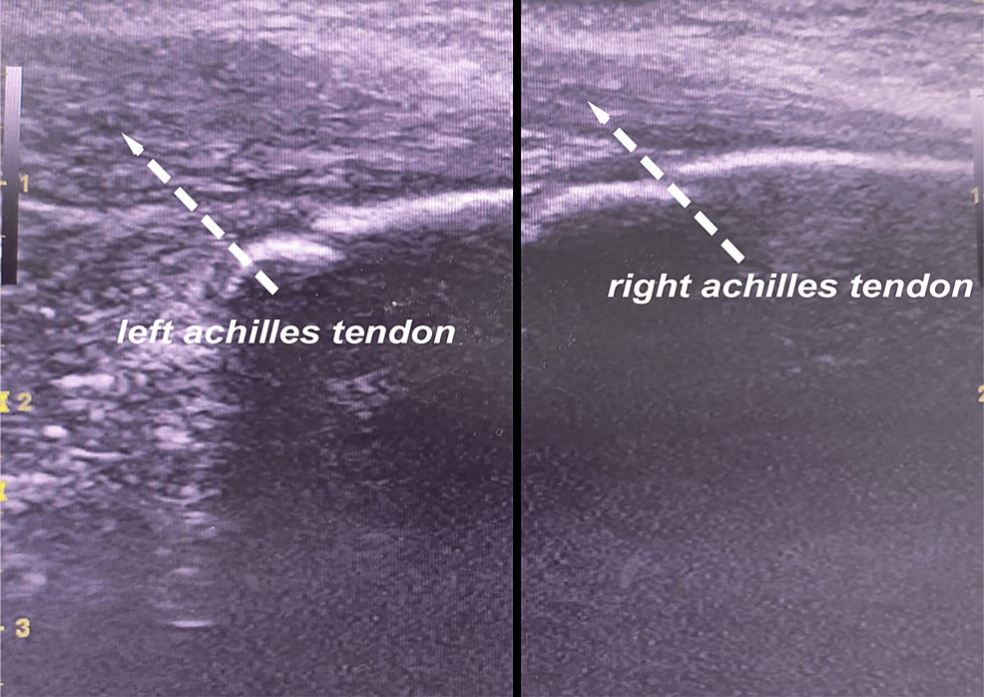
Comparative ultrasound section of the heel demonstrating thickening of the Achilles tendon on the left side with no associated retrocalcaneal bursitis

Comparative diagnostic ultrasound of the heel (Figures 2-3) displayed a fragmented appearance of the calcaneal apophysis, along with thickening of the left Achilles tendon in comparison to the unaffected side. Notably, no tendon tears were observed, but a positive Doppler signal was detected. Importantly, the imaging findings showed no signs of retrocalcaneal bursitis or plantar fasciitis.

The patient received a diagnosis of Sever's disease and was prescribed oral anti-inflammatory medication (400 mg of oral ibuprofen, three times a day) along with topical diclofenac to be applied to the heels for a duration of three weeks. Furthermore, the patient was advised to use heel raise shoe orthoses and avoid engaging in sports activities that may exacerbate the condition. Given the coexistence of Achilles tendinitis and Sever's disease, an extended treatment period of 10 weeks was recommended.

The patient was educated on stretching and strengthening exercises and was instructed to undergo twenty sessions of stretching, along with 15 minutes of cold compresses applied to the left heel. Manual stretching and joint mobilization techniques were also administered. After 10 weeks of therapy, a follow-up examination revealed a reduction in complaints. No sensitivity was detected upon palpation of the heels, and the joints exhibited a normal range of motion. There were no observed gait abnormalities. Consequently, the patient was cleared to resume their daily and sports activities.

## Discussion

The most important finding of the study was the successful management of Sever's disease in the presented case through a multimodal approach. Calcaneal apophysitis, commonly known as Sever's disease, represents a prevalent cause of heel pain in active children. This condition predominantly affects physically active individuals within the age range of eight to 15 years [3].

Mechanical overuse resulting from repetitive impact pressure and shear stresses on the open growth plate of the calcaneus constitutes the principal hypothesis for the pathogenesis of Sever's injury [3]. Elevated levels of physical activity and obesity emerge as the predominant risk factors contributing to the development of Sever's disease in pediatric patients [4]. Furthermore, a research study identified a significant association between calcaneal apophysitis and higher body mass index, increased weight, and greater height among affected individuals [5].

During the physical examination, tenderness is observed upon palpation and compression at the medial and lateral aspects of the heel. Erythema or swelling of the heel is typically absent. Limited ankle joint dorsiflexion is commonly observed, and physical activity tends to exacerbate the pain. Clinical diagnosis can be confirmed by performing a "squeeze test," which involves applying medial and lateral compression to the heel, resulting in pain [3].

Radiographic findings associated with Sever's injury commonly include two distinct observations: increased density and fragmentation of the calcaneal apophysis. These radiographic features serve as evidence of the condition and aid in its diagnosis [6]. However, these two radiographic findings are not specific and do not exclusively indicate the diagnosis of Sever's injury [6].

Radiographic plain films are essential in the evaluation of pediatric patients with heel pain to exclude other potential causes. Fractures, dislocations, bone cysts in the calcaneus, osteoid osteoma, and various tarsal bone conditions can present with similar symptoms and should be ruled out. These imaging studies help provide a comprehensive assessment and ensure an accurate diagnosis of Sever's disease by excluding other possible etiologies [7].

In children with suspected Sever's apophysitis, an MRI was conducted to confirm the diagnosis [8]. The MRI findings revealed edema in the fatty marrow of the apophysis, and in severe cases, involvement of the adjacent body of the calcaneus and heel pad. These pathological changes are most clearly visualized using short tau inversion recovery (STIR) or fat-suppressed T2-weighted sagittal or transverse sequences [8]. MRI serves as a valuable tool in assessing the extent and severity of the inflammatory process, providing crucial information for accurate diagnosis and appropriate management of Sever's disease [8].

In the majority of cases, radiological examinations consistently demonstrate the absence of abnormalities in the Achilles tendon when utilizing ultrasound for the assessment of the calcaneal apophysis [9]. Ultrasound examination reveals various pathologic findings associated with Sever's disease, including pretibial swelling, fragmentation of the ossification center, insertional thickening of the patellar tendon, and excessive fluid collection in the infrapatellar bursa, ultrasound serves as a straightforward and reliable diagnostic tool for identifying these abnormalities [10]. This underscores the specificity of ultrasound in detecting and differentiating the pathologic changes related to Sever's disease [10].

The treatment of Sever's disease involves a comprehensive approach that encompasses various modalities. This includes the use of ice therapy, restriction of physical activity, stretching exercises, administration of nonsteroidal anti-inflammatory drugs (NSAIDs) such as ibuprofen, immobilization, and the use of heel cups to alleviate pain associated with Sever's disease [11]. These interventions and treatments have been extensively studied to assess their efficacy in managing the condition. It is important to note that pain in Sever's disease is closely linked to activity and sports participation; therefore, modifying or reducing physical activities during painful episodes is advised as part of the treatment plan [11]. The application of taping to the foot, specifically around the arch and heel area, has demonstrated effectiveness in reducing pain associated with Sever's disease. Arch taping has been found to have a positive impact on alleviating pain related to Sever's disease and improving ambulation [11].

Incorporating calf muscle stretching exercises into the treatment plan is recommended as it provides direct traction on the Achilles tendon. These exercises aim to strengthen specific muscles, thereby reducing the workload on the Achilles tendon [12]. In severe cases or when dealing with noncompliant patients, immobilization through the use of a boot or cast may be necessary. These devices are typically worn for a period of two to four weeks and are often combined with rehabilitation therapies to optimize the treatment outcome [13].

The recovery period for Sever's disease can vary among children, and it is influenced by factors such as the extent of activity restriction and adherence to other treatment modalities. It is important to note that full recovery is typically achieved once a child reaches skeletal maturity [2].

## Conclusions

In summary, this case report underscores the successful management of Sever's disease in a 10-year-old patient through a comprehensive approach. Key findings include confirmation of the diagnosis via clinical examination and ultrasound, revealing characteristic features such as pretibial swelling and ossification center fragmentation. These ultrasound findings are vital for accurate diagnosis. The multimodal treatment approach resulted in significant improvement after 10 weeks. Our study emphasizes the importance of early recognition, accurate diagnosis, and multimodal management for successful outcomes in Sever's disease.
